# Survival Status and Predictors of Mortality Among Women With Breast Cancer in Southern Ethiopia: A Retrospective Cohort Study

**DOI:** 10.1002/cnr2.70722

**Published:** 2026-10-16

**Authors:** Bitsiet Desta, Tsion Zebdiwos Chema, Sophonias Sisay, Mulualeme Nigusie, Bargude Balta

**Affiliations:** ^1^ College of Health Science Wolaita Sodo University Sodo Ethiopia; ^2^ College of Health Science Hawassa University Hawassa Ethiopia

**Keywords:** breast cancer, predictors, southern Ethiopia, survival

## Abstract

**Introduction:**

Breast cancer continues to be a leading cause of morbidity and mortality worldwide, with an especially severe impact in low‐resource settings. In such contexts, delayed diagnosis, limited diagnostic capacity, and inadequate access to treatment contribute to poor survival outcomes. However, little is known about how these factors influence survival patterns specifically in southern Ethiopia, highlighting the need for locally generated data to guide interventions.

**Methodology:**

A hospital‐based retrospective cohort study was conducted among 507 breast cancer patients attending selected hospitals in southern Ethiopia. Data were collected by two trained BSc nurses using an Open Data Kit (ODK) tool. Descriptive statistics were presented using tables and graphs, while bivariable, and multivariable Cox regression models were used to identify independent predictors of survival.

**Results:**

A total of 507 medical records were reviewed, of which 492 eligible women were included in the final analysis. The overall survival rates were 54.5% at 2 years and 23.9% at 3 years. In multivariable Cox regression analysis, advanced stage had a higher hazard of death (AHR = 1.73, 95% CI: 1.19–2.51). Poor NPI was also associated with increased mortality (AHR = 2.30, 95% CI: 1.50–5.10). Patients with an NLR < 3 had a lower hazard of death (AHR = 0.68, 95% CI: 0.47–0.97), as did those who received hormonal therapy (AHR = 0.64, 95% CI: 0.44–0.92).

**Conclusion:**

Advanced‐stage disease and poorer NPI were associated with a higher hazard of death from any cause, while lower NLR and hormonal therapy were associated with better overall survival. Strengthening early diagnosis and ensuring appropriate treatment may improve overall survival among women with breast cancer in resource‐constrained settings.

**Abbreviations**
AHR: adjusted hazard ratio
BC: breast cancer
CHR: crude hazard ratio
GBCI: global breast cancer initiative
HIV: human immunodeficiency virus
LMIC: low‐ and middle‐income countries
MSF: metastasis‐free survival
NLR: neutrophil‐to‐lymphocyte ratio
NPI: Nottingham Prognostic Index
ODK: open data kit
OS: overall survival

## 1 Background

Breast cancer remains a major global public health challenge. In 2022, approximately 2.3 million women were diagnosed with breast cancer worldwide, making it the second most frequently occurring cancer overall, while approximately 666 000 deaths were attributed to the disease, placing it fourth among all causes of cancer mortality. Among women, breast cancer is the most frequently diagnosed malignancy and remains the leading cause of cancer‐related mortality in many countries [1, 2, 3]. The burden of breast cancer varies considerably across regions and is strongly influenced by socioeconomic development and the availability of cancer care services.

Although countries with higher human development generally report higher incidence, women in low‐ and middle‐income countries experience substantially poorer outcomes. Sub‐Saharan Africa is particularly affected, with high breast cancer mortality relative to incidence. Limited access to screening and early detection, delayed diagnosis, inadequate diagnostic capacity, and restricted availability of appropriate treatment contribute substantially to these unfavorable outcomes [1, 4]. In addition, urbanization and changes in dietary and lifestyle patterns may contribute to the increasing burden of breast cancer in developing countries [5].

The burden of breast cancer is also increasing in Ethiopia. Current estimates indicate an incidence rate of approximately 40.8 per 100 000 women and a mortality rate of 24.0 per 100 000 [6]. Data from the Addis Ababa Cancer Registry indicate that breast cancer accounts for approximately one‐third of cancers diagnosed among women and nearly one‐quarter of all registered cancers [7]. Despite efforts to expand cancer services, access to essential components of breast cancer management, including surgery, radiotherapy, diagnostic services, and specialized nursing care, remains limited in many settings [2, 8]. These limitations contribute to substantial differences in survival between high‐income and resource‐constrained countries. Five‐year survival exceeds 90% in many high‐income countries but has been reported to be approximately 66% in India and 40% in South Africa [2].

Considerable variation in survival has also been reported across African countries. Previous studies have documented 5‐year survival rates of approximately 58% in Sudan and 74% in Malawi [9, 10]. Evidence from Ethiopia similarly demonstrates substantial heterogeneity, with reported five‐year overall survival estimates ranging from 25.8% to 72.1% [6, 7, 11, 12]. These differences may reflect variation in stage at diagnosis, access to diagnostic and treatment services, tumor characteristics, and patient‐level factors.

Breast cancer survival is influenced by a combination of demographic, tumor‐related, clinical, and treatment‐related characteristics. Sociodemographic factors, including age, residence, and educational status, have been associated with survival outcomes [11, 13, 14]. Tumor‐related characteristics such as tumor size, lymph‐node involvement, histological grade, disease stage, and comorbid conditions have also been identified as important prognostic factors [10, 15]. Biological characteristics, including estrogen and progesterone receptor status and HER2/neu expression, further contribute to differences in prognosis [16, 17]. In addition, clinical and treatment‐related factors, including the neutrophil‐to‐lymphocyte ratio (NLR), chemotherapy, and surgical treatment, have been associated with breast cancer survival [6, 18, 19, 20, 21].

The World Health Organization (WHO), through its Global Breast Cancer Initiative (GBCI), promotes a comprehensive approach centered on health promotion and early detection, timely diagnosis, and comprehensive breast cancer management. The initiative aims to reduce global breast cancer mortality by 2.5% annually, with the potential to avert 2.5 million breast cancer deaths over 20 years [22]. However, achieving these targets remains challenging in resource‐limited settings because of shortages in diagnostic infrastructure, treatment capacity, trained personnel, and other essential cancer‐care resources [22, 23, 24, 25, 26, 27, 28, 29, 30].

Although reducing modifiable risk factors is important, prevention alone is unlikely to eliminate the majority of breast cancer cases. Even substantial reductions in established modifiable risk factors may prevent only a proportion of breast cancer occurrence [1, 31, 32, 33, 34, 35]. Therefore, strengthening health systems, expanding access to early detection and timely diagnosis, and improving the availability and quality of treatment are essential components of breast cancer control, particularly in low‐ and middle‐income countries where cancer services remain insufficient and unevenly distributed [23, 36, 37, 38, 39, 40, 41].

Although several studies have investigated breast cancer survival in Ethiopia, much of the available evidence has originated from Addis Ababa and the northern and western parts of the country. Evidence from southern Ethiopia remains comparatively limited. Moreover, few studies have examined the prognostic significance of readily available clinical measures such as the Nottingham Prognostic Index (NPI) and NLR within the same patient population. These indicators may provide useful information for risk stratification in routine oncology settings, particularly where access to advanced prognostic investigations is limited. Regional differences in disease presentation, diagnostic pathways, treatment availability, and patient characteristics further emphasize the need for locally generated evidence.

Therefore, this study aimed to determine survival outcomes and identify predictors of mortality among women with breast cancer in southern Ethiopia. By evaluating both established clinicopathological characteristics and accessible prognostic indicators, the study seeks to provide evidence that can inform clinical risk stratification, treatment prioritization, and breast cancer control strategies in resource‐limited settings.

## 2 Methods

### 2.1 Study Area and Period

The study was conducted in Hawassa City and Wolaita Sodo, located approximately 273 and 365 km south of Addis Ababa, respectively. Hawassa University Comprehensive Specialized Hospital (HUCSH), located in Hawassa City, has a 650‐bed capacity and provides both curative and palliative care services to patients from the Sidama Region and neighboring areas. The oncology unit is staffed by 25 health professionals, including 14 bachelor's degree nurses, 2 general practitioners, 2 oncologists, 2 oncology nurses, 2 laboratory technicians, and 3 pharmacists.

Wolaita Sodo University Comprehensive Specialized Hospital, located in Wolaita Sodo, has a 420‐bed inpatient capacity and provides both curative and palliative care services to patients from Wolaita and surrounding areas. Its oncology unit is staffed by 13 health professionals, including 8 bachelor's degree nurses, 4 general practitioners, 1 oncology nurse, and 2 oncologists. The study was conducted from March 1 to March 30, 2023.

### 2.2 Study Design and Population

This retrospective cohort study was conducted using medical records of women with breast cancer who received care at Hawassa University Comprehensive Specialized Hospital and Wolaita Sodo University Comprehensive Specialized Hospital in southern Ethiopia. Women aged 18 years or older who had a confirmed diagnosis of breast cancer between January 1, 2020 and December 31, 2022 were considered eligible for inclusion. The source population comprised women diagnosed with breast cancer in Ethiopia, whereas the study population consisted of eligible patients whose records were available at the participating hospitals during the study period. Participants were followed from the date of breast cancer diagnosis until death, loss to follow‐up, or the end of the observation period, whichever occurred first.

The present investigation used the same underlying cohort previously described in a study that examined the prognostic significance of the NLR among women with breast cancer in southern Ethiopia [42]. Although the participant cohort and observation period overlap, the objectives and analytical focus of the present study are distinct. Specifically, the previous study evaluated the association between NLR and breast cancer prognosis, whereas the present study focuses on overall survival and identifies a broader range of demographic, clinical, pathological, and treatment‐related predictors of mortality, including the NPI. The use of the same cohort is therefore explicitly acknowledged to ensure transparency and to distinguish the present analysis from the previously published work [43].

### 2.3 Exclusion Criteria

Clients with incomplete records regarding baseline clinical characteristics (stage, tumor size, and nodule status), unknown outcome status, and no telephone response were excluded from the study. Clients whose medical charts were lost were also excluded from the study.

### 2.4 Sample Size Determination

The minimum sample size required with an exponential model in Stata 16, considering the following assumptions: Type I error rate of 5%, power of 80%, and premenopausal status (AHR = 1.33) [6], was 461. After adding a 10% contingency for missing and incomplete data, the sample size for this study was 507.

### 2.5 Sampling Procedure

The sampling frame was prepared after collecting the medical registration or Identification number of patients from the registration books or the electronic database of each hospital. Based on the number of women who received treatment, the sample size was proportionally allocated among the hospitals. By using proportionate allocation, 183 and 324 women with breast cancer were selected from WSUCSH and HUCSH, respectively, giving a total of 507 medical records. Finally, the study participants were selected by a systematic random sampling technique based on their respective kth values.

### 2.6 Study Variables

The dependent variable of this study was time to death, defined as the duration (in months) from the date of first confirmed breast cancer diagnosis to death from any cause during the follow‐up period. The independent variables included socio‐demographic, tumor‐related, and clinical/treatment‐related factors. Socio‐demographic variables comprised age at diagnosis, place of residence, educational status, family history of breast cancer, and menopausal status. Tumor‐related predictors included tumor size, axillary lymph node involvement, histological grade, and stage at diagnosis. Clinical and treatment‐related variables included comorbidity status, type of initial treatment, chemotherapy, surgical treatment, radiotherapy, hormone therapy, NLR, and diagnosis‐to‐treatment interval (DTI).

### 2.7 Operational Definitions

#### 2.7.1 Event

An event was defined as death occurring after the first confirmed diagnosis of breast cancer and before the end of the study follow‐up period.

#### 2.7.2 Censored

Censoring referred to patients who were alive at the end of the follow‐up period, lost to follow‐up, or discharged with medical advice before experiencing the event.

#### 2.7.3 Adherence to Chemotherapy

Chemotherapy adherence was operationally defined as the proportion of prescribed chemotherapy cycles actually received by a patient, based on chemotherapy prescription and administration records. Patients who received ≥ 80% of their prescribed chemotherapy cycles were classified as adherent, whereas those who received < 80% were classified as non‐adherent [44, 45].

#### 2.7.4 DTI

DTI was defined as the time (in days) between the date of histopathological confirmation of breast cancer and the initiation of the first definitive cancer treatment.

#### 2.7.5 NLR

NLR was calculated by dividing the absolute neutrophil count by the absolute lymphocyte count obtained from baseline complete blood count measurements before treatment initiation.

#### 2.7.6 Breast Cancer Stage

Breast cancer staging was classified according to the Tumor–Node–Metastasis (TNM) staging system.

- Early‐stage breast cancer was defined as TNM Stages I and II.
- Advanced‐stage breast cancer was defined as TNM Stages III and IV, including locally advanced and metastatic disease.

#### 2.7.7 NPI

The NPI was calculated using the formula: *NPI = [tumor size (cm) × 0.2] + lymph node stage* [1, 2, 3] *+ histological grade* [1, 2, 3].

Based on the NPI score, patients were categorized as follows:

- Excellent prognosis: ≤ 2.4
- Good prognosis: > 2.4 to ≤ 3.4
- Moderate prognosis: > 3.4 to ≤ 5.4
- Poor prognosis: > 5.4 [20, 21]

### 2.8 Data Collection Procedures

An English‐language data extraction checklist was developed following a review of relevant literature [6, 11, 14, 41]. The checklist was structured to capture sociodemographic, tumor‐related, clinical, treatment‐related, and outcome information. Sociodemographic variables included age at diagnosis, residence, educational status, family history of breast cancer, and menopausal status. Tumor characteristics included tumor size, lymph‐node involvement, histological grade, disease stage, and estrogen and progesterone receptor status. Clinical and treatment variables comprised comorbidity, initial treatment modality, surgery, chemotherapy, radiotherapy, hormonal therapy, and NLR. The primary outcome was overall survival, with participants classified according to their status at the end of follow‐up as deceased, alive, or lost to follow‐up.

Eligible records of women diagnosed with breast cancer between January 1, 2020 and December 31, 2022 were identified through the cancer registries of the participating hospitals. Information was subsequently obtained from patient medical records, electronic databases, operative notes, and other relevant clinical documentation using the structured extraction checklist. Both baseline and subsequent follow‐up records were examined to obtain complete information on disease characteristics, treatment, and vital status.

Follow‐up commenced on the date of confirmed breast cancer diagnosis and continued until death, loss to follow‐up, the last documented contact, or the administrative end of follow‐up, whichever occurred first. Vital status was established primarily from medical records and, where necessary, through telephone contact with patients or their families. For patients reported to have died, available documentation, including death certificates and hospital records, was reviewed to verify the outcome. Two trained BSc nurses extracted the data electronically using Open Data Kit (ODK) Collect. The completed records were reviewed for completeness and consistency before analysis.

### 2.9 Data Quality Assurance

A pre‐test was done on 5% of the study sample; patient charts were checked for the existence of variables in the registration format on the patient's chart. Before the data collection period, 1 day of training was given to data collectors and supervisors. On‐site supervision was given to solve any doubts about data collection tools and techniques. The collected information was checked for consistency and completeness on the same day by the principal investigator. Reviewed cards were boldly marked to avoid re‐review. Data cleaning was checked against the codebook for any missing values and data errors.

### 2.10 Data Analysis

Data were collected using ODK Collect version 1.25.2 and exported to STATA version 16 for analysis. Descriptive statistics, including frequencies, proportions, medians, tables, and charts, were used to summarize the characteristics of the study participants. Kaplan–Meier survival analysis was used to estimate overall survival probabilities, and differences in survival between groups were assessed using the log‐rank test. A Cox proportional hazards regression model was used for the analysis of factors associated with time to death. The Cox model was selected because it does not require specification of the baseline hazard distribution. The proportional hazards assumption was assessed using Schoenfeld residuals and the global test. Multicollinearity among the independent variables was assessed before fitting the multivariable model using variance inflation factor (VIF) and tolerance statistics. A VIF < 10 and tolerance > 0.10 were considered to indicate no problematic multicollinearity. Particular attention was given to the simultaneous inclusion of cancer stage and NPI. Variables with a *p* value < 0.25 in the bivariable analysis were entered into the multivariable Cox regression model. Crude and adjusted hazard ratios (CHR and AHR) with 95% confidence intervals were reported, and statistical significance was declared at *p* < 0.05. The final analysis was based exclusively on the Cox proportional hazards model; the previously considered Weibull analysis was not retained in the final model. Estrogen/progesterone receptor and HER2 status were not included in the multivariable survival analysis because these variables were available for only 243 (49.4%) of the 492 participants, resulting in substantial missing data. Therefore, these variables were excluded from the multivariable model to avoid loss of a substantial proportion of the study population.

## 3 Results

### 3.1 Sociodemographic Characteristics

Of the 507 patient medical records reviewed, 492 breast cancer patient charts met the inclusion criteria and were included in the analysis, yielding a completeness rate of 97%. More than half (54.27%) of the study's participants were women between the ages of 35 and 50, with a median age of 40 and an interquartile range (IQR) of 34 to 49 years. The majority, 290 (58.94%) of the women, were rural residents, and 138 (25.05%) of them had never attended school. More than a quarter (26.42%) of women were postmenopausal (Table 1).

**TABLE 1:** Sociodemographic characteristics of breast cancer women in southern Ethiopia, 2023.

Characteristics	*n*	%
Age		
< 35 years	125	25.41
35–50 years	267	54.27
50 + years	100	20.33
Residency		
Urban	202	41.06
Rural	290	58.94
Educational status		
No formal education	138	28.05
Primary school	164	33.33
Secondary school	127	25.81
Above secondary	63	12.80
Marital status		
Single	47	9.55
Married	354	71.95
Divorced	50	10.16
Widowed	41	8.33
Menopausal status		
Premenopausal	362	73.58
Postmenopausal	130	26.42

### 3.2 Clinical, Histopathological, and Treatment Characteristics

Among the 492 women included in the study, 314 (63.82%) had advanced‐stage breast cancer, while 178 (36.18%) had early‐stage disease. Distant metastasis was present in 253 (51.42%) patients and positive nodal status in 241 (48.98%). According to the NPI, 225 (45.70%) patients were classified in the poor‐prognosis category. Ductal carcinoma was the predominant histological type (390, 79.27%), while 102 (20.73%) had non‐ductal carcinoma.

Breast surgery was performed in 177 (35.98%) patients, among whom 122 (68.9%) had free and 55 (31.1%) had involved deep surgical margins. Chemotherapy was administered to 417 (84.76%) patients, while 221 (44.92%) received hormonal therapy and 22 (4.47%) received radiation therapy. Recurrence occurred in 110 (22.4%) patients. Regarding the diagnosis‐to‐treatment interval, 210 (42.68%) patients initiated treatment within 1 month of diagnosis, whereas 282 (57.32%) had a DTI of 1 month or longer (Table 2).

**TABLE 2:** Clinical, histopathological, and treatment characteristics of Breast cancer in southern Ethiopia, 2023.

Characteristics	*n*	%
Cancer stage at diagnosis		
Early	178	36.18
Advanced	314	63.82
Co‐morbidities		
Yes	90	18.29
No	402	81.71
NLR		
1–3	327	66.46
> 3	165	33.54
NPI		
Excellent	76	15.45
Good	53	10.77
Moderate	138	28.00
Poor	225	45.70
Histological type		
Ductal	390	79.27
Non‐ductal carcinoma	102	20.73
Distant metastasis		
Yes	253	51.42
No	239	48.58
Nodal status		
Positive	241	48.98
Negative	251	51.02
Surgical therapy		
Yes	177	35.98
No	315	64.02
Surgery type (*n* = 177)		
MRM	120	67.8
Toilet mastectomy	12	6.77
Partial mastectomy	33	18.64
Others	12	6.77
Recurrence status		
Yes	110	22.4
No	382	77.6
Deep surgical margin		
Free	122	68.9
Involved	55	31.1
Chemotherapy		
Yes	417	84.76
No	75	15.24
*NACT*		
Yes	122	29.3
No	295	70.7
Adherence to chemotherapy (*n* = 417)		
Good	85	20.38
Poor	332	79.62
Hormonal status (*n* = 243)		
Positive	149	61.3
Negative	94	38.7
HER2 status (*n* = 243)		
Positive	132	54.3
Negative	111	45.7
Hormonal therapy		
Yes	221	44.92
No	271	55.08
Hormonal therapy type		
Tamoxifen	167	75.57
Anastrozole	54	24.43
Radiation therapy		
Yes	22	4.47
No	470	95.53
DTI		
< 1 month	210	42.68
≥ 1 month	282	57.32

Abbreviations: DTI: diagnosis to treatment initiation; NLR: ratio of neutrophils to lymphocytes; NPI: Nottingham Prognostic Index.

### 3.3 Survival Status of Patients With Breast Cancer

A total of 492 women with breast cancer were followed for a maximum of 36 months. Among the 492 women included in the final analysis, 152 (30.9%; 95% CI: 26.8–35.0) died from any cause during the follow‐up period, 283 (57.5%; 95% CI: 53.1–61.8) were alive at the end of follow‐up, and 57 (11.6%; 95% CI: 8.8–14.5) were lost to follow‐up. The total time at risk accumulated by the 492 study participants was 5634.9 person‐months. The median survival time was 28.1 (95% CI: 23.2–30.8) months. Overall survival was 71.8% at 1 year, 54.5% at 2 years, and 23.9% at 3 years (Figure 1).

**FIGURE 1:**
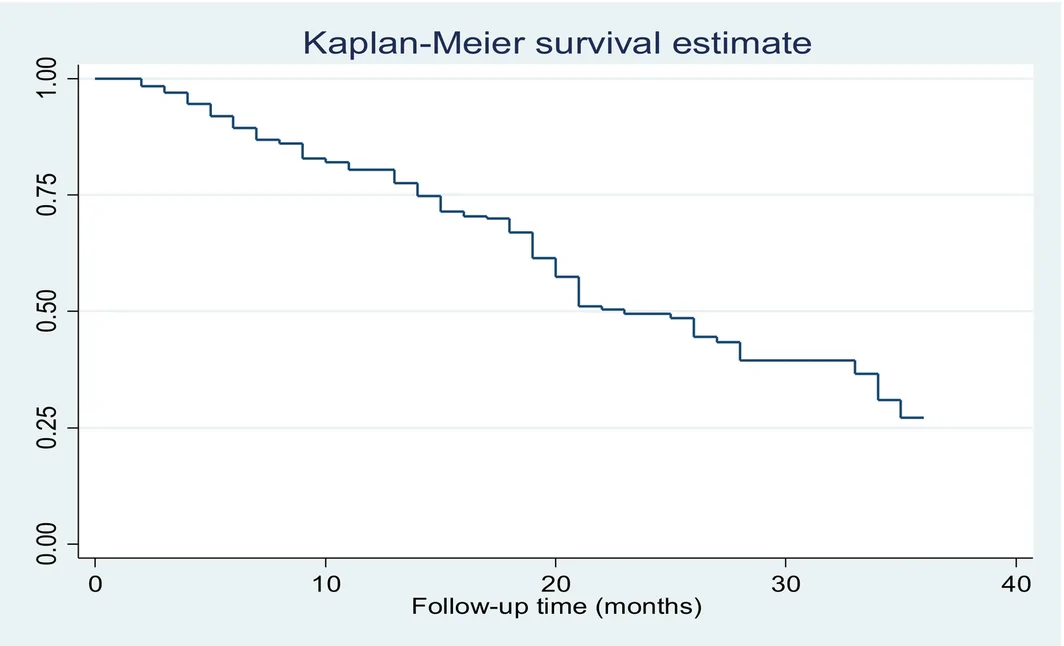
Plot of the overall estimate of Kaplan–Meier survivor function of breast cancer patients in southern Ethiopia, 2023.

The incidence rate of death from any cause was 3.1 per 100 person‐months among women who initiated treatment at an early stage and 3.7 per 100 person‐months among those who initiated treatment at an advanced stage (Figure 2).

**FIGURE 2:**
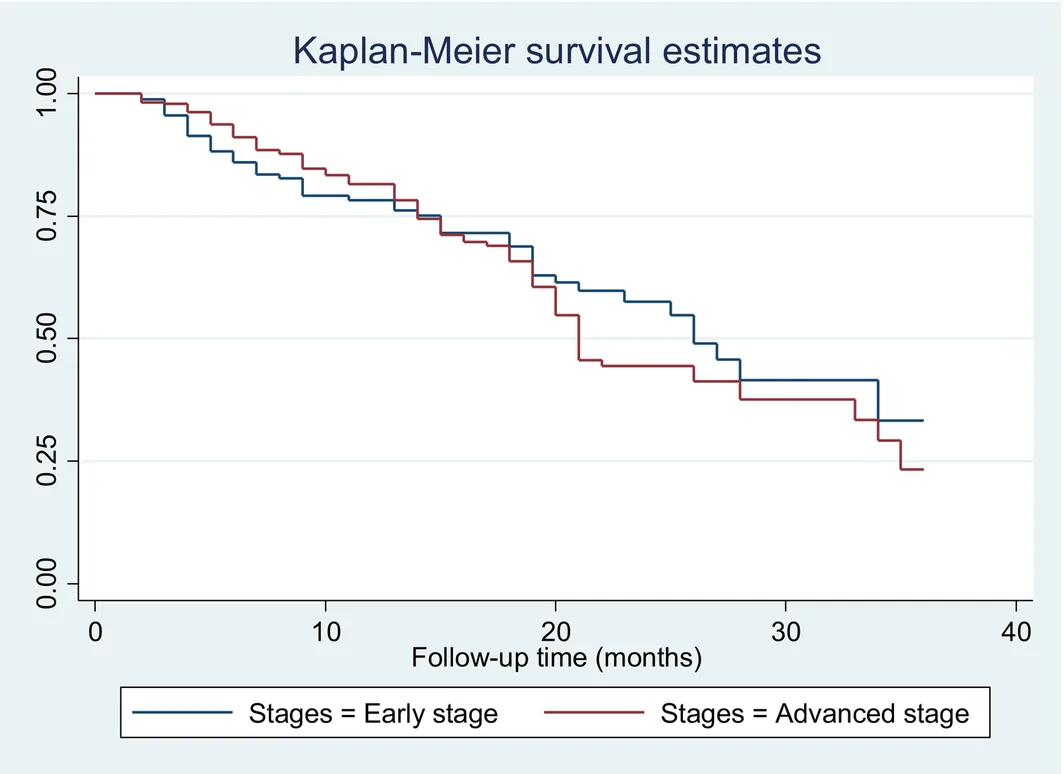
Kaplan–Meier survival estimates based on stage of breast cancer, southern Ethiopia, 2023.

The incidence rate of death from any cause among breast cancer patients with a poor NPI was 5.6 per 100 person‐months, compared with 2.5 and 0.5 per 100 person‐months among those with moderate and excellent NPI, respectively (Figure 3).

**FIGURE 3:**
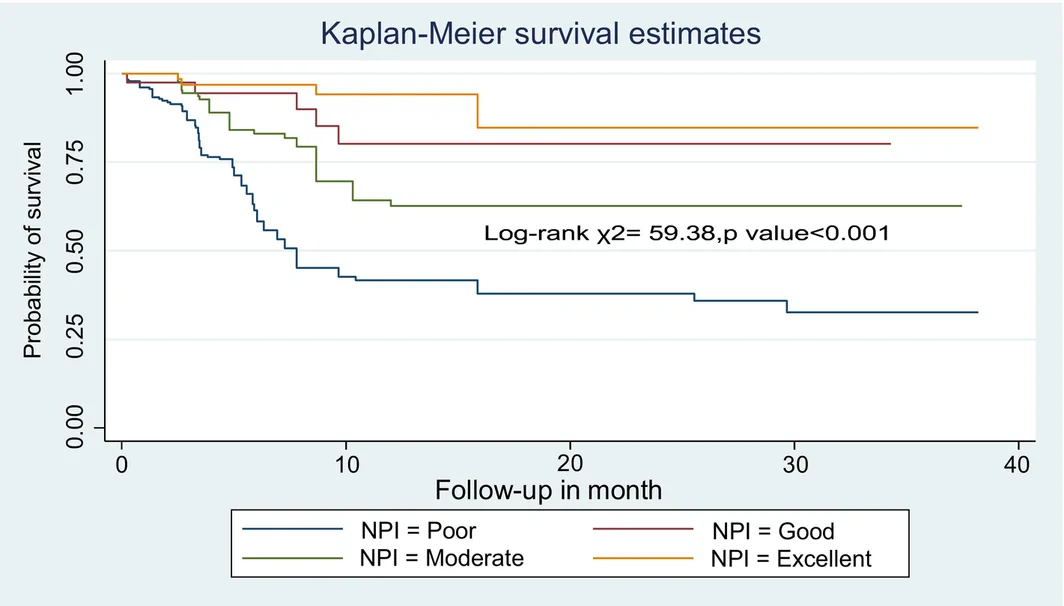
Kaplan–Meier survival estimates based on Nottingham prognostic index for breast cancer in southern Ethiopia, 2023.

### 3.4 Bivariate and Multivariate Analysis of Breast Cancer Patients' Survival in Southern Ethiopia

In the multivariable Cox proportional hazards regression analysis, advanced‐stage breast cancer, moderate and poor NPI categories, a NLR of < 3, and hormonal therapy were independently associated with overall survival.

#### 3.4.1 Model Diagnostics

The proportional hazards assumption was satisfied, as the global Schoenfeld residual test was not statistically significant (*p* = 0.297). There was no evidence of problematic multicollinearity, with VIF values ranging from 1.23 to 3.30 and tolerance values ranging from 0.51 to 0.90.

Patients with advanced‐stage disease had a significantly higher hazard of death from any cause compared with those with early‐stage disease (AHR = 1.73, 95% CI: 1.19–2.51). Compared with patients with an excellent NPI, those with a moderate NPI had a higher hazard of death from any cause (AHR = 1.20, 95% CI: 1.01–4.20), while those with a poor NPI had more than twice the hazard of death from any cause (AHR = 2.30, 95% CI: 1.50–5.10). Patients with an NLR < 3 had a lower hazard of death from any cause than those with an NLR ≥ 3 (AHR = 0.68, 95% CI: 0.47–0.97). Similarly, patients who received hormonal therapy had a lower hazard of death from any cause compared with those who did not receive hormonal therapy (AHR = 0.64, 95% CI: 0.44–0.92). Age, place of residence, menopausal status, educational status, surgical margin, histology type, breast surgery, chemotherapy, and diagnostic‐to‐treatment interval were not significantly associated with overall survival after adjustment for other covariates, as their 95% confidence intervals included unity (Table 3).

**TABLE 3:** Bivariable and multivariable Cox regression analysis of predictors of breast cancer survival among women in southern Ethiopia, 2023.

Characteristics	Event *n* (%)	Censored *n* (%)	CHR (95% CI)	AHR (95% CI)
Age (years)				
< 35 years	32 (25.6)	93 (74.4)	1	1
35–50 years	83 (31.1)	184 (68.9)	1.30 (0.86–1.96)	1.18 (0.76–1.82)
> 50 years	37 (37.0)	63 (63.0)	1.38 (0.85–2.21)	1.26 (0.78–2.05)
Place of residence				
Urban	64 (31.7)	138 (68.3)	1	1
Rural	88 (30.3)	202 (69.7)	1.10 (0.80–1.52)	1.06 (0.77–1.47)
Menopausal status				
Premenopausal	98 (27.1)	264 (72.9)	1	1
Postmenopausal	54 (41.5)	76 (58.5)	1.30 (0.92–1.80)	1.21 (0.84–1.74)
Educational status				
No formal education	59 (42.8)	79 (57.3)	1	1
Primary	47 (28.7)	117 (71.3)	0.79 (0.54–1.16)	0.86 (0.57–1.29)
Secondary	32 (25.2)	95 (74.8)	0.54 (0.35–0.84)	0.71 (0.43–1.17)
College & above	14 (22.2)	49 (77.8)	0.66 (0.37–1.20)	0.79 (0.42–1.49)
Stage of breast cancer				
Early stage	44 (24.7)	134 (75.3)	1	1
Advanced stage	108 (34.4)	206 (65.6)	1.72 (1.21–2.44)	1.73 (1.19–2.51)*
Nottingham Prognostic Index (NPI)				
Excellent	16 (21.1)	60 (78.9)	1	1
Good	8 (15.1)	45 (84.9)	0.67 (0.25–0.82)	0.70 (0.35–1.32)
Moderate	38 (27.5)	100 (72.5)	1.43 (1.01–2.40)	1.20 (1.01–4.20)*
Poor	90 (40.0)	135 (60.0)	2.50 (1.70–5.80)	2.30 (1.50–5.10)*
Neutrophil‐to‐lymphocyte ratio (NLR)				
< 3	90 (27.5)	237 (72.5)	0.67 (0.48–0.92)	0.68 (0.47–0.97)*
≥ 3	62 (37.6)	103 (62.4)	1	1
Surgical margin				
Free	35 (28.2)	87 (71.3)	1	1
Involved	20 (36.4)	36 (63.6)	1.24 (0.89–1.73)	1.15 (0.82–1.63)
Histology type				
Ductal	119 (30.5)	271 (69.5)	0.89 (0.60–1.30)	0.94 (0.63–1.41)
Non‐ductal carcinoma	33 (32.4)	69 (67.6)	1	1
Breast surgery				
Yes	46 (26.0)	131 (74.0)	0.79 (0.56–1.12)	0.88 (0.61–1.27)
No	106 (33.7)	209 (66.3)	1	1
Chemotherapy				
Yes	122 (29.3)	295 (70.7)	0.62 (0.41–0.93)	0.78 (0.50–1.20)
No	30 (40.0)	45 (60.0)	1	1
Hormonal therapy				
Yes	69 (31.2)	152 (68.8)	0.64 (0.44–0.92)	0.64 (0.44–0.92)*
No	83 (30.6)	188 (69.4)	1	1
Diagnostic treatment interval (DTI)				
< 1 month	60 (28.6)	150 (71.4)	1	1
≥ 1 month	92 (32.6)	190 (67.4)	1.03 (0.81–1.30)	1.08 (0.84–1.39)

*Note:* Variables with *p*‐value ≤ 0.25 in bivariable analysis were entered into the multivariable Cox regression model. Statistically significant at *p* < 0.05.

Abbreviations: AHR = adjusted hazard ratio; CHR = crude hazard ratio; CI = confidence interval; NLR = neutrophil‐to‐lymphocyte ratio; NPI = Nottingham Prognostic Index.

## 4 Discussion

This study assessed overall survival and prognostic factors among women with breast cancer treated at selected hospitals in southern Ethiopia. The estimated overall survival was 54.5% at 2 years and decreased to 23.9% at 3 years. After adjustment for potential confounding factors, disease stage, NLR, hormonal therapy, and NPI remained independently associated with overall survival.

The 3‐year survival observed in the present study was lower than survival estimates reported from northwest Ethiopia (54.2%) and western Ethiopia (53%) [6, 23]. However, the survival estimate was also lower than the 5‐year survival reported in studies from Sudan (58%), China (64.42%), Vietnam (74%), Turkey (82.3%), Iran (87%), Brazil (87.3%), France (87.6%), and the United Arab Emirates (89%) [3, 9, 17, 28, 30, 35, 40, 46]. These differences should be interpreted cautiously because survival estimates are influenced by the duration of follow‐up, study design, population characteristics, stage distribution, treatment availability, and methods used to estimate survival.

Differences in access to timely diagnosis and comprehensive cancer treatment may also contribute to the poorer outcomes observed in resource‐limited settings. However, comparisons across settings should be interpreted cautiously because of differences in follow‐up duration, stage at diagnosis, availability of diagnostic and treatment services, and patient and tumor characteristics. More importantly, our findings should be considered within the broader regional context reported by Parenté et al. [47], who synthesized evidence from 85 studies involving 33 053 patients across sub‐Saharan Africa and reported a pooled 5‐year survival of 44%, with a pooled survival of 48% in East Africa.

In the present study, 63.82% of women were diagnosed at an advanced stage, only 4.47% received radiotherapy, 57.32% experienced a DTI of 1 month or longer, and 79.62% of patients who received chemotherapy had poor treatment adherence. These findings suggest substantial gaps in timely and comprehensive breast cancer care that may partly explain the poor survival observed in this cohort. This interpretation is supported by Gnangnon et al. [42], who reported important gaps in the continuum of breast cancer care across 47 hospitals in 15 sub‐Saharan African countries; only 55.3% of hospitals had a full‐time pathologist, 19.1% had radiotherapy services, 53.1% had chemotherapy drugs available on‐site, and only 6.4% met all minimum criteria for both diagnosis and treatment.

These regional constraints provide an important context for the low uptake of surgery, limited radiotherapy use, and treatment delays observed in our study. Differences in stage at diagnosis, availability of diagnostic and treatment services, treatment completion, and patient and tumor characteristics may also contribute to variations in survival across settings. Strengthening early detection, timely diagnosis, pathology and surgical services, radiotherapy capacity, treatment completion, and access to comprehensive breast cancer care is therefore important for improving survival outcomes in Ethiopia.

Hormonal therapy was associated with improved overall survival in this cohort, with women receiving endocrine therapy experiencing a 36% lower hazard of death from any cause compared with those who did not receive hormonal treatment. Similar findings have been reported in previous studies from Ethiopia and other settings [24, 48].

This survival benefit is likely attributable to the effectiveness of endocrine therapy among hormone receptor‐positive patients rather than a comparison between receptor‐positive and receptor‐negative groups. However, this association should be interpreted with caution because hormone receptor status was incomplete for many patients, and information on treatment adherence and access to care was limited. Therefore, residual confounding may have influenced the observed relationship, as patients receiving hormonal therapy may have differed systematically from those who did not in unmeasured clinical characteristics. The limited availability of HER2 and hormone receptor testing reflects ongoing diagnostic resource constraints in many Ethiopian oncology settings and highlights the need to strengthen pathology and biomarker testing services to support evidence‐based breast cancer management.

Advanced stage at diagnosis was independently associated with poorer overall survival. Women presenting with advanced‐stage breast cancer had a 1.73‐fold higher hazard of death from any cause compared with those diagnosed at an early stage (AHR = 1.73; 95% CI: 1.19–2.51). This finding is consistent with reports from Northwest Ethiopia and other low‐resource settings [6, 48]. Advanced disease was also associated with poorer survival outcomes, with patients having metastatic breast cancer experiencing a higher hazard of death from any cause compared with those without metastasis. This finding is consistent with previous studies from China and northern Brazil [18, 24]. The presence of advanced stage indicates advanced systemic disease and is associated with limited treatment options, higher disease burden, and reduced likelihood of long‐term survival [49, 50, 51]. Advanced‐stage disease is often associated with greater tumor burden, lymph node involvement, and distant spread, which contribute to treatment challenges and reduced survival. The higher hazard of death observed among patients with advanced disease emphasizes the importance of early detection, timely diagnosis, and improved access to effective treatment services.

The NPI was a strong independent predictor of overall survival in this study. Compared with patients with an excellent NPI, those with moderate and poor NPI had higher hazards of death from any cause. In particular, poor NPI was associated with more than twice the hazard of death from any cause (AHR = 2.30, 95% CI: 1.50–5.10). Similar findings have been reported in previous studies demonstrating the prognostic value of NPI in breast cancer survival prediction [52]. The association between higher NPI scores and poorer outcomes may reflect the combined influence of tumor size, lymph node involvement, and histological characteristics, which are important determinants of disease progression and treatment response.

A lower NLR was independently associated with improved overall survival. Patients with an NLR < 3 had a 32% lower hazard of death from any cause compared with those with higher NLR values (AHR = 0.68; 95% CI: 0.47–0.97). Similar associations have been reported in studies from China and Turkey [18, 46, 53]. Elevated NLR reflects systemic inflammation and an imbalance between neutrophil‐mediated tumor progression and lymphocyte‐mediated antitumor immune responses, which may contribute to aggressive disease behavior and poorer outcomes. These findings suggest that NLR may serve as a simple and cost‐effective prognostic marker, particularly in resource‐limited settings where advanced biomarker testing is not consistently available.

The present study used the same underlying cohort as a previous BMJ Open study [43], which examined the prognostic value of the NLR. However, the present analysis addresses a broader research question by evaluating overall survival and multiple demographic, clinical, pathological, and treatment‐related predictors of death from any cause using multivariable Cox proportional hazards regression.

This study was limited by several factors. First, the retrospective cohort design relied on routinely recorded medical information, which may be subject to information bias due to missing, incomplete, or inaccurately documented clinical data. Second, the exclusion of patients with incomplete records may have introduced selection bias if excluded patients had different characteristics or outcomes from those included in the analysis. Third, the absence of complete hormone receptor and HER2 status data limited our ability to perform molecular subtype‐specific analyses. In addition, some deaths occurring outside healthcare facilities may not have been captured, and loss to follow‐up may have resulted in underestimation or overestimation of overall survival outcomes. Future prospective studies should record complete receptor status and molecular subtype data to enable more robust subgroup analyses.

## 5 Conclusion

This study provides important regional evidence on overall survival and prognostic factors among women with breast cancer in southern Ethiopia, where survival outcomes remain poor. Advanced‐stage disease and poorer NPI categories were independently associated with a higher hazard of death from any cause, while a lower NLR and receipt of hormonal therapy were associated with better overall survival. The findings suggest that NPI and NLR may have potential value as accessible prognostic markers for identifying patients at higher risk of death, particularly in resource‐constrained settings. Strengthening early detection and timely diagnosis, ensuring appropriate treatment, and optimizing the use of recommended breast cancer therapies, including hormonal therapy where clinically indicated, may contribute to improved survival outcomes. These findings may inform clinical decision‐making and breast cancer control strategies in Ethiopia and similar low‐ and middle‐income settings.

Future prospective studies and implementation research are needed to further evaluate the prognostic value of NPI and NLR and to assess interventions aimed at improving early diagnosis, timely treatment, and appropriate delivery of evidence‐based breast cancer therapies. Regular monitoring of overall survival following implementation of such strategies will be important to evaluate their effectiveness in improving survival outcomes among women with breast cancer in Ethiopia.

## Author Contributions

**Bitsiet Desta:** conceptualization, investigation, validation, visualization, funding acquisition, writing – original draft, writing – review and editing, formal analysis, data curation, resources. **Tsion Zebdiwos Chema:** data curation, supervision, resources, project administration, software. **Sophonias Sisay:** investigation, writing – original draft, visualization, writing – review and editing, software, formal analysis, data curation. **Mulualeme Nigusie:** conceptualization, investigation, writing – original draft, writing – review and editing. **Bargude Balta:** resources, supervision, data curation, writing – review and editing, visualization, methodology, conceptualization, funding acquisition, writing – original draft.

## Funding

The authors have nothing to report.

## Ethics Statement

The Research and Ethics Committee (REC) of the School of Nursing and Midwifery, College of Health Sciences, Addis Ababa University, granted ethical approval (Ref. No. 08/SNM/15). The respective hospital authorities granted permission to access the patients' medical records. For participants whose vital status could not be established from the medical records, telephone contact with patients or their family members was used when necessary, as part of the approved follow‐up procedures. The study was conducted in accordance with the principles of the Declaration of Helsinki.

## Consent

The REC waived the requirement for informed consent for the retrospective review and follow‐up procedures. All patient information was anonymized and kept strictly confidential.

## Conflicts of Interest

The authors declare no conflicts of interest.

## Data Availability

The data that support the findings of this study are available on request from the corresponding author. The data are not publicly available due to privacy or ethical restrictions.
